# Development and validation of a psychometric scale for assessing pharmacy students’ perceptions and attitudes toward antimicrobial resistance and antimicrobial stewardship in Indonesia: the PATARAS study

**DOI:** 10.1186/s12909-025-07375-5

**Published:** 2025-05-30

**Authors:** Ikhwan Yuda Kusuma, Ria Benko, Muh. Akbar Bahar, Dian Ayu Eka Pitaloka, Soeharto Soeharto, Doni Anshar Nuari, Rani Prabandari, Dezső Csupor, Mária Matuz

**Affiliations:** 1 Institute of Clinical Pharmacy, Faculty of Pharmacy, University of Szeged, Szeged, 6725 Hungary; 2 Pharmacy Study Program, Faculty of Health, Universitas Harapan Bangsa, Purwokerto, 53182 Indonesia; 3 Institute of Clinical Pharmacy, University of Szeged, Albert Szent-Györgyi Health Centre, Szeged, 6725 Hungary; 4 Department of Pharmacy, Faculty of Pharmacy, Universitas Hasanuddin, Makassar, 90245 Indonesia; 5 Department of Pharmacology and Clinical Pharmacy, Faculty of Pharmacy, Universitas Padjadjaran, Sumedang, 45363 Indonesia; 6 Center of Excellence in Higher Education for Pharmaceutical Care Innovation, Universitas Padjadjaran, Sumedang, 45363 Indonesia; 7 Doctoral School of Educational Sciences, Faculty Humanities and Social Science, University of Szeged, Szeged, 6722 Hungary; 8 Department of Pharmacy, Faculty Mathematics and Natural Science, Universitas Garut, Garut, 44151 Indonesia; 9 Research Center of Educational Technologies, Azerbaijan State University of Economics, Baku, Azerbaijan; 10 Research Center for Education, National Research and Innovation Agency (BRIN), Jakarta, Indonesia

**Keywords:** Antimicrobial, Perception, Attitude, Pharmacy students, SEM

## Abstract

Appropriate perceptions and attitudes of pharmacists toward antimicrobial resistance (AMR) are essential. However, AMR attitudes and perceptions have not been assessed in Indonesia. This study aimed to develop and validate a questionnaire to evaluate pharmacy students’ Perceptions and Attitudes toward Antibiotic Resistance and Antimicrobial **S**tewardship (PATARAS) and investigate the relationship between students’ perceptions and attitudes toward antibiotic resistance and stewardship. Using purposive sampling, 500 undergraduate pharmacy students were included in the study. The 20-item PATARAS questionnaire with 5-point Likert scales was developed. The PATARAS includes perception (rational drug use (RDU) and antimicrobial stewardship (AMS)) and attitude (professional role (PRO) and inappropriate practice (IPP)) domains. The validity (convergent and discriminant) of the PATARAS was investigated using confirmatory factor analysis (CFA). The CFA model fit was assessed using various goodness-of-fit indices. Reliability was assessed using Cronbach’s alpha (α) and McDonald’s Omega (ω) values. The relationship between subdomains was evaluated using structural equation modeling (SEM). The PATARAS questionnaire achieved acceptable convergent and discriminant validity. The CFA model achieved a good fit. The reliability was good with α and ω values of 0.817 and 0.845, respectively. The SEM showed that RDU and AMS were positively correlated with PRO (β = 0.121 and 0.539, respectively, *p* < 0.05); RDU and IPP were also positively correlated (β = 0.313, *p* < 0.05), while AMS and IPP were negatively correlated (β = − 0.275, *p* < 0.05). The PATARAS is a valid and reliable instrument for assessing undergraduate pharmacy students’ perceptions and attitudes toward AMR and AMS.

**Supplementary Information:**

The online version contains supplementary material available at 10.1186/s12909-025-07375-5.

## Introduction

As health care professionals, pharmacists play a crucial role in supplying information about drugs (both prescription and over-the-counter) to patients [1]. Knowledge gained from graduate pharmacy education that includes antimicrobial stewardship (AMS) contributes to the optimization of antimicrobial therapy. The World Health Organization (WHO) has published a curriculum guide on antibiotic resistance for health care teaching and training, which includes pharmacy education [2]. Reviews have also been published on AMS, including the content and methods for delivering AMS [3, 4]. However, no study specifically examined the relationship between perceptions and attitudes about antibiotics, antimicrobial resistance (AMR), or AMS in pharmacy students. Previous studies focused on the perception and attitudes of pharmacy students toward antibiotic use, such as those conducted in Sri Lanka [5] and Saudi Arabia [6], however, these studies did not specifically address AMR or AMS as distinct and structured constructs. Studies assessing antibiotic use, AMR, and AMS in pharmacy students were conducted in Pakistan [7] and the UK [8],however, these studies did not comprehensively assess the relationship between perception and attitude.

The perceptions and attitudes regarding AMR and AMS are established before pharmacists enter daily practice. Thus, the development of a standardized instrument for measuring these parameters is urgently needed. The present study aimed to develop and validate a new Perceptions and Attitudes Toward Antimicrobial Resistance and Stewardship (PATARAS) questionnaire and investigate the relationship between perceptions and attitudes toward AMR and AMS among Indonesian pharmacy students.

## Methods

A cross-sectional study was conducted in Indonesia from February to May 2022. This study was divided into three phases: (1) PATARAS questionnaire development, five experts (four pharmacy lecturers and one education expert) reviewed the items for face and content validity; (2) Pilot testing, 30 undergraduate pharmacy students participated in item testing and refinement.; and (3) Confirmatory Factor Analysis and Structural Equation Modeling, investigating the relationship between perceptions and attitudes of pharmacy students toward AMR and AMS using the final version of the questionnaire, administered to 500 undergraduate pharmacy students, whose characteristics are presented in Table 1.

**Table 1:** Characteristics of study respondents (*n* = 500)

Description	Frequency (*n* = 500)	Percentage (%)
**Gender**		
Female	425	85%
Male	75	15%
**Age**		
< 20 years old	117	35.4%
20–23 years old	294	58.8%
> 23 years old	29	5.8%
**Semester**		
2nd (1st year study)	130	26.0%
4th (2nd year study)	152	30.4%
6th (3rd year study)	121	24.2%
8th (4th year study)	90	18%
10th (5th year study)	6	1.2%
12th (6th year study)	1	0.2%
**Workplace Goals**		
Community Pharmacist	59	11.8%
Hospital Pharmacist	201	40.2%
Government Pharmacist	47	9.4%
Industrial Pharmacist	166	33.2%
Educational Pharmacist	11	2.2%
Health Center Pharmacist	14	2.8%
Other	2	0.4%

### PATARAS development

The PATARAS questionnaire was developed in five steps: (1) framework development, (2) item generation, (3) item screening (face and content validity), and (4) pilot test, (5) validation and structural modeling [9, 10].

#### Framework development

The theoretical framework was built using content analysis of literature and included socio-demographics as the background and two domains toward antibiotics: perception and attitude. Two subdomains were set for each domain. The perception domain consisted of rational drug use (RDU) and AMS, while the attitude domain consisted of professional roles (PRO) and inappropriate practices (IPP). Perception was defined as students’ understanding and awareness related to antibiotic use, AMR, and AMS principles. Attitude referred to students’ willingness, professional attitude, and behavioral intentions toward antibiotic-related responsibilities. Antimicrobial Stewardship (AMS) was defined as students’ perception of coordinated strategies aimed at optimizing antimicrobial use to combat resistance. Professional Role (PRO) encompassed the sense of responsibility and readiness of students to engage in antibiotic stewardship as part of their future pharmacy roles. Inappropriate Practice (IPP) refers to students' agreement with or tendency toward behaviors such as self-medication, stockpiling antibiotics, or dispensing without proper indications. The following publications were used to develop a comprehensive framework related to the perception and attitude toward antibiotics among pharmacy students: 1) a practical handbook published by WHO for developing knowledge, attitude, and practice surveys [11]; 2) a psychology conceptual consensus for applying evidence-based practice [12]; 3) previous studies using questionnaires about pharmacy students’ perceptions and attitudes toward antibiotic used [6–8].

#### Item generation

Item generation was performed to identify items that fit the domain [9]. A comprehensive review of the literature and content analysis of existing questionnaires was conducted to determine the domain content that we wished to assess and include in the PATARAS questionnaire [8, 13, 14]. Item generation involved identifying items, answer possibilities, and scoring methods [15]. The literature review resulted in the identification of 20 items, which were assessed using a five-point Likert scale from strongly agree (5) to strongly disagree (1), for the two domains and four subdomains (Table 2). The instrument was originally developed simultaneously in both Bahasa Indonesia and English, as it was designed for bilingual use among pharmacy students and academic settings in Indonesia. Therefore, forward–backward translation procedures were not required.

**Table 2:** Convergent validity and reliability test result

Domain	Subdomain	Code	Items	Loading Factor	Reliability	
					**Cronbach's α**	**McDonald's ω**
Perception	Rational Drug Use (RDU)	RDU 1	I received sufficient pharmacy education about antibiotics and infectious diseases	0.776	0.887	0.891
		RDU 2	I received sufficient pharmacy education to select the appropriate regimen (dose, route, frequency) for antibiotic therapy)	0.817		
		RDU 3	I received sufficient pharmacy education to identify patients who request antibiotic without prescription without clinical indications and to provide accurate and appropriate information about the risks of antibiotic use	0.678		
		RDU 4	I received sufficient pharmacy education to understand the mechanism of antibiotic resistance	0.694		
		RDU 5	I received sufficient pharmacy education to interpret the results of antibiotic susceptibility test	0.715		
		RDU 6	I received sufficient pharmacy education to choose the appropriate combination of antibiotics	0.824		
		RDU 7	I received sufficient pharmacy education about Antimicrobial Stewardship during my education in pharmacy	0.623		
	Antimicrobial Stewardship (AMS)	AMS 1	I interested to receive more education on antibiotic use, resistance, and stewardship to contribute the AMS program in the future	0.673	0.677	0.724
		AMS 2	Antimicrobial education among health students can help to minimize the phenomenon of antibiotic resistance	0.828		
		AMS 3	Antibiotic resistance is a significant clinical issue in my current and future professional career	0.524		
Attitude	Professional Role (PRO)	PRO 1	All pharmacists are required to provide pharmaceutical care related to antibiotics use	0.696	0.769	0.779
		PRO 2	I am willing to combat antibiotic resistance and participate in the Antimicrobial Stewardship team as part of my responsibilities as a pharmacist, even without special incentives	0.736		
		PRO 3	I want to be a prepared pharmacist to contribute to combat antibiotic resistance	0.759		
		PRO 4	Pharmacist need to take responsibility for rational antibiotics use	0.537		
	Inappropriate Practice (IPP)	IPP 1	Antibiotics are relatively safe, these drugs can be commonly used to treat infections disease	0.678	0.829	0.838
		IPP 2	When my family members get ill, I usually give them antibiotics stored from previous leftover treatments	0.945		
		IPP 3	I normally keep antibiotic stocks at home in case of emergency	0.750		
Total Reliability Statistics All Factor (RDU. AMS. PRO. IPP)					0.817	0.845

A loading factor is a correlation coefficient that represents the strength of the relationship between a particular measure or item and the subdomain that it represents

#### Item screening (Face and Content Validity)

The content validity of a questionnaire refers to how well the items sample the domain of interest. The expert panel’s review (face validity by experts) and selection of the most appropriate items were based on the content validity [9, 16]. The expert panel from the research team was composed of four pharmacy lecturers and one education expert. The panel discussed the suitability of each item by assessing the clarity, concreteness, relevance, and importance in measuring the perception and attitude toward antibiotics for each item. To quantitatively assess item relevance, the Content Validity Index (CVI) method was employed. Twenty draft items were independently rated by five experts using a 4-point relevance scale. Item-level CVI (I-CVI) and scale-level CVI (S-CVI) were calculated, with an I-CVI threshold of ≥ 0.78 applied for item retention [17]. Feedback was provided on wording clarity, domain alignment, and redundancy. Items not meeting the threshold were flagged for exclusion, and minor revisions were applied to improve clarity and consistency before pilot testing. This process ensured that retained items were representative of each subdomain (RDU, AMS, PRO, and IPP) and understandable to the target population. The experts finalized the questionnaire items, identified the essential items, excluded non-essential items, and modified items as required [15].

#### Pilot test

A pilot test was performed for the PATARAS questionnaire to assess the clarity and difficulty of each item (polishing items by students) [10]. A total of 30 undergraduate pharmacy students participated in the pilot test [15, 18]. Participants were requested to submit corrections and suggestions to generate an understandable and relevant questionnaire [19]. Preliminary internal consistency was assessed using Cronbach’s alpha, with each domain exceeding the minimum acceptable threshold of 0.6 [20]. No items were deleted solely based on pilot test reliability. Assessment of dimensionality confirming the PATARAS questionnaire structure and the overall factor structure was performed during the main validation phase using confirmatory factor analysis (CFA). The pilot test results were refined and included in the final questionnaire for validation.

### Data collection

The participants included 500 undergraduate bachelor of pharmacy students (education program before professional pharmacist education) who were purposively chosen from 90 universities in Indonesia. To ensure wide representation, we used purposive sampling by leveraging the Association of Indonesian Pharmacy Higher Education/the Asosiasi Pendidikan Tinggi Farmasi Indonesia (APTFI), which comprises 161 institutions [21]. APTFI is divided into five regional coordination forums (Forum Wilayah, ForWil: (1 Sumatra; (2 West Java–Banten–DKI Jakarta–West Kalimantan; (3) Central Java–Yogyakarta–South Kalimantan; (4) East Java–Bali–Nusa Tenggara; and (5) East Kalimantan–North Kalimantan–Sulawesi–Maluku–Papua [21]. Through coordination with ForWil representatives, academic lecturers from each region were invited to assist in distributing the questionnaire. Participating universities were selected based on willingness to collaborate and regional diversity. Each university was encouraged to nominate 2 to 6 undergraduate students across academic years to complete the survey via a Google Form.

This study focused on undergraduate students because, according to the Indonesian pharmacy curriculum, foundational topics related to antibiotics and infectious diseases are typically introduced between the second and fourth semesters. Including students from all years enabled us to capture the development of perceptions and attitudes across different stages of academic progression. Pharmacy students in higher education are future professional pharmacists who will play a critical role in the healthcare workforce and medical education. This approach aligns with the Knowledge-Attitude-Practice (KAP) framework, which theorizes that knowledge influences attitudes and behavior. Furthermore, it supports FIP Development Goal 17, which emphasizes the importance of initial education in equipping the pharmaceutical workforce with competencies related to antimicrobial stewardship [22]. A direct message or email with a Google Form link was distributed to students via faculty lecturers to ensure wide representation.

### Validation and reliability

#### Construct validity

Confirmatory factor analysis (CFA) was used to assess the construct validity (convergent and discriminant validities) of the perception and attitude items included in the questionnaire. Convergent validity is the extent that items converge into the same subdomain or items produce similar results, while subdomain analysis is a statistical method used to identify underlying factors or dimensions that explain the relationships among multiple subdomains (e.g. the relationship between RDU and AMS) [9]. The loading factor is the correlation between an item and the subdomain (e.g. RDU, AMS, PRO, or IPP) that it represents. A measurement with high convergent validity is expected to have a high loading factor score for the subdomains that it represents [23, 24]. When the items within the subdomains are highly correlated, the measure has convergent validity. A loading factor of at least 0.5 is considered acceptable [25].

Discriminant validity statistically evaluates the degree to which two measurements are distinct and do not overlap in the measurement of subdomains. A low correlation coefficient implies that distinct subdomains are measured and indicates high discriminant validity [24]. The correlation coefficient ranges from − 1 to 1; negative values indicate a negative correlation, 0 indicates no correlation, and 1 indicates a perfect positive correlation [24]. Items should significantly impact only one domain. The correlation coefficient should not exceed 0.7. A correlation greater than 0.7 indicates a large shared variance. Moreover, if “cross-loading” exists (variable loads on multiple factors), then cross-loading should differ by more than 0.240. Several indices were used to assess how well the CFA model fit the data, including the comparative fit index (CFI): > 0.90; the root mean squared error of approximation (RMSEA): ≤ 0.08; the standardized root mean squared residual (SRMSR): ≤ 0.08; and the Tucker–Lewis index (TLI): > 0.92.

#### Reliability

The reliability of the PATARAS was assessed using Cronbach’s alpha (α) and McDonald’s Omega (ω) [26], which measure the consistency and stability of the questionnaire’s results. To achieve high reliability, all items within the questionnaire should be relevant and assess a particular subdomain. Respondents’ scores on the measured items should positively correlate, indicating that the questionnaire items are internally consistent. Cronbach's alpha and McDonald’s omega should be above 0.6 [11, 27]. When reliability coefficients (α or ω) were below 0.6, we performed item-total correlation analysis to identify weak items (correlation < 0.3). These items were then reviewed by an expert panel and either revised or removed to improve internal consistency and conceptual clarity [20, 28].

This study only focuses on confirming validity, reliability and investigate the relationship between subdomain, therefore no specific method was used to calculate composite score for each subdomains. All the outputs or values were generated based on SEM analysis using Jamovi software. As this study focused on instrument development and validation, no cut-off values were applied to classify responses (e.g., into "positive" or "negative" categories). Future research may establish interpretive thresholds using normative data, percentile-based classifications, or receiver operating characteristic (ROC) curve analysis to support applied decision-making or intervention design as recommended by Streiner et al. [29].

### SEM modeling

The relationship between subdomains was determined using SEM modeling (Fig. 2). We hypothesized that perceptions (RDU and AMS) directly affect attitudes (PRO and IPP) toward AMR and AMS. R^2^ values were also determined to understand the variance among subdomains based on the structural model. R^2^ values measured the total variance for each endogenous (dependent) variable shown in the center of exogenous (independent) variables in Fig. 2 and also measured the explanatory and predictive power of constructs in the models [30, 31]. A value as low as 0.10 is considered satisfactory, depending on the specific conditions [32].

### Statistical analysis

Descriptive statistics were used to summarize participant characteristics and are presented as numbers and percentages. Analyses were performed using SPSS version 26 (IBM [33]). The PATARAS questionnaire was validated using CFA and SEM to provide the path analysis and run using JAMOVI version 2.3 [26].

## Results

### Face and content validity

Twenty items were initially identified as eligible for inclusion in the PATARAS questionnaire. The expert panel scrutinized the PATARAS questionnaire draft and removed three items, resulting in 17 items in the final questionnaire. The three items were eliminated because they were repetitive, irrelevant, or imprecise, as outlined in Appendix 1. Students were invited to provide feedback on the readability and comprehensibility of the PATARAS questionnaire. Based on their feedback, all questionnaire items were deemed readable and understandable.

### Participants

The 17 items of the PATARAS questionnaire were completed by 500 undergraduate bachelor of pharmacy students from 90 Indonesian universities via the online data collection platform. Out of 895 invitations sent, 500 students responded and completed the PATARAS questionnaire, resulting in an overall response rate of approximately 55.9%, which is acceptable for large-scale, online, education-based survey research [34]. On average, each participating university contributed around 5 to 6 students. This sample reflects wide institutional and regional representation, covering public and private universities from all five APTFI regional coordination forums across Indonesia. The demographic characteristics of the respondents are summarized in Table 1. A majority of students (*n* = 425; 85%) were female, and 58.8% of respondents were 20–23 years old (*N* = 294). A majority of respondents (*N* = 152, 30.4%) were in their fourth semester. Most of the participants (*N* = 201, 40.2%) preferred to be a pharmacist in a hospital after graduation.

### Convergent validity

**Fig. 1:**
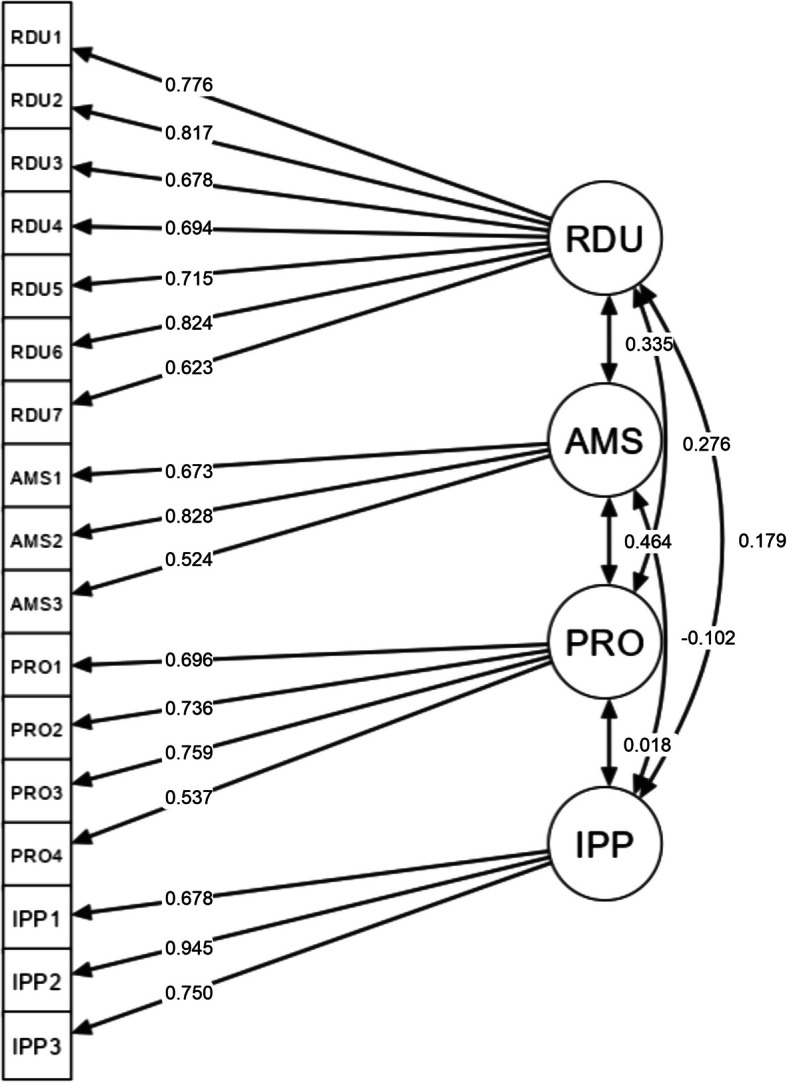
Confirmatory factor analysis diagram. The loading factor value is depicted by the direction of the arrow toward each item, and the discriminant validity values were based on the correlation values for each subdomain. RDU: Rational Drug Use; AMS: Antimicrobial Stewardship; PRO: Professional Role; IPP: Inappropriate Practice

The loading factor values for the four subdomains in the PATARAS questionnaire (perception domain: RDU and AMS; attitude domain: PRO and IPP) ranged from 0.524 to 0.945 (Fig. 1). Thus, all loading factors meant the convergent validity requirements (Table 2).

### Discriminant validity

All subdomains in the PATARAS questionnaire achieved the discriminant validity threshold (Table 3). The measures of rational drug use (RDU), Antimicrobial Stewardship (AMS), Professional Role (PRO), and inappropriate practice (IPP) had different levels of correlation, but items in each construct had acceptable loading factors above 0.5. Thus, all items converged into specific subdomains.

**Table 3:** Correlation matrix (Discriminant validity)

	**RDU**		**AMS**		**PRO**		**IPP**
RDU	—						
AMS	0.335	***	—				
PRO	0.276	***	0.464	***	—		
IPP	0.176	***	-0.102	*	0.018	*	—

*RDU* Rational Drug Use, *AMS* Antimicrobial Stewardship, *PRO* Professional Role, *IPP* Inappropriate Practice

^*^ *p* < .05. ** *p* < .01. *** *p* < .001

### Reliability

The reliability assessment of subdomains in the PATARAS questionnaire showed acceptable values for Cronbach’s alpha, ranging from 0.677 to 0.887, and McDonald’s omega, ranging from 0.724 to 0.891. The overall reliability values of the PATARAS questionnaire were acceptable, with a Cronbach's alpha and McDonald’s omega of 0.821 and 0.844, respectively.

### Model fit statistics of CFA

Based on the CFA results, the model achieved an acceptable threshold of fit with the data (CFI = 0.929; TLI = 0.915; SRMR = 0.049; RMSEA = 0.067). Therefore, the PATARAS exhibited excellent model fit criteria.

### Structural model using SEM modeling

**Fig. 2:**
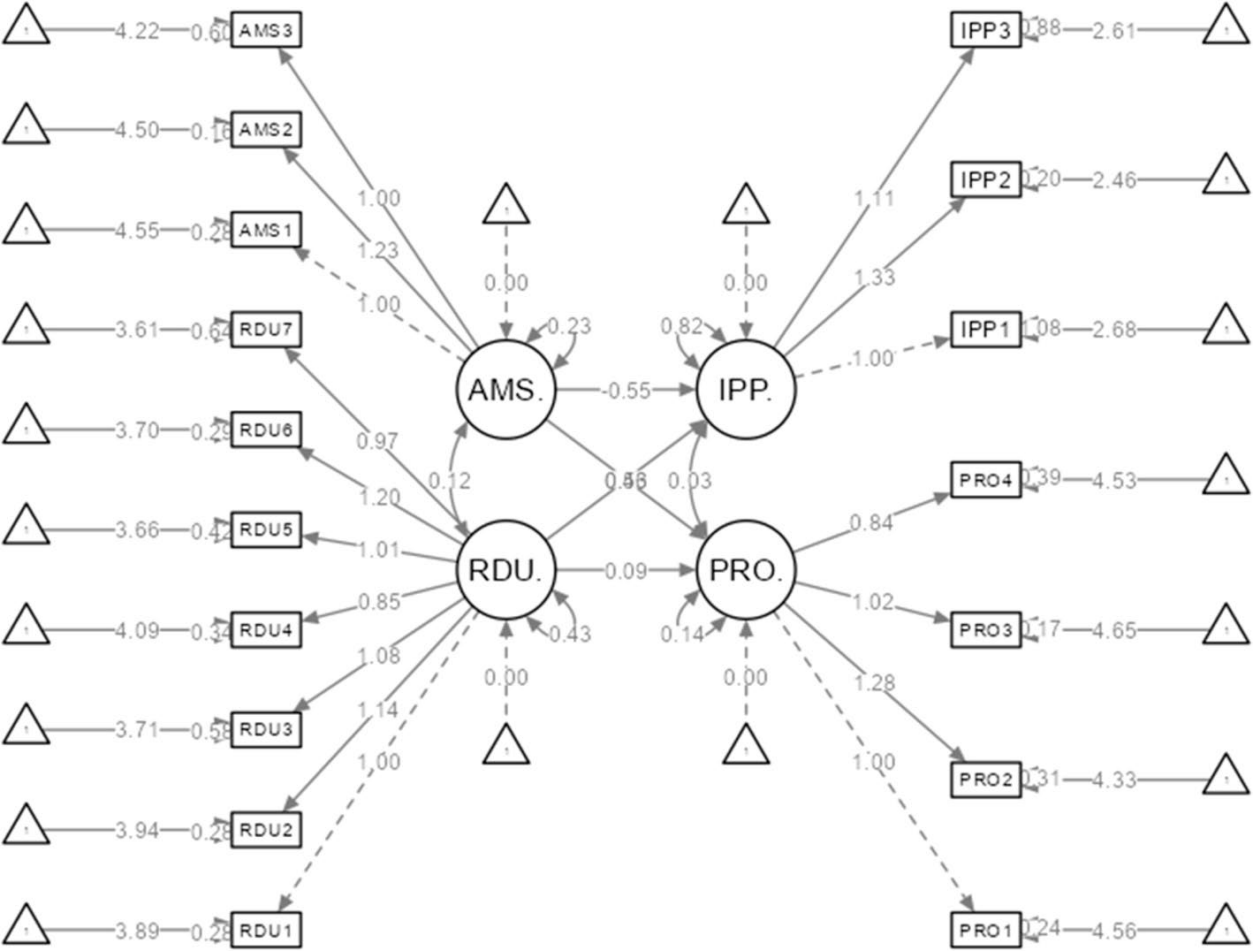
Structural equation modeling diagram for the PATARAS subdomains. Circles indicate subdomains or constructs, squares indicate manifest variables (item indicators), and triangles indicate errors, or residuals [35]. RDU: Rational Drug Use; AMS: Antimicrobial Stewardship; PRO: Professional Role; IPP: Inappropriate Practice

SEM modeling was performed to investigate the relationship between the subdomains, as shown in Fig. 2.

Assessment of the SEM shows that the direct effects of RDU and AMS on PRO and IPP were statistically significant. RDU and AMS positively correlated with PRO (β = 0.121 and 0.539, respectively, *p* < 0.05). Thus, the higher perception of RDU and AMS, the better the attitude toward the PRO. RDU and IPP also positively correlated (β = 0.313, *p* < 0.05); thus, a higher perception of RDU lowers IPP such as dispensing antibiotics over the counter for every infectious disease. Lastly, AMS and IPP negatively correlated (β** = ** − 0.275, *p* < 0.05), indicating that a higher perception of AMS lowers IPP. A summary of the path coefficients is shown in Table 4. The two endogenous constructs (dependent variables), PRO, and IPP, had R^2^ values of 0.354 and 0.108, respectively.

**Table 4:** Summary of finding according to path coefficients in the structural model

					**95% CI**				
**Independent**		**Dependent**	**Estimate**	**SE**	**Lower**	**Upper**	**β**	**z**	**p**
RDU	→	PRO	0.0868	0.0382	0.0119	0.162	0.121	2.27	0.023
RDU	→	IPP	0.4574	0.0837	0.2934	0.621	0.313	5.47	< .001
AMS	→	PRO	0.5298	0.0674	0.3977	0.662	0.539	7.86	< .001
AMS	→	IPP	-0.5486	0.1220	-0.7877	-0.310	-0.275	-4.50	< .001

*CI* Confidence Intervals, *RDU* Rational Drug Use, *AMS* Antimicrobial Stewardship, *PRO* Professional Role, *IPP* Inappropriate Practice

## Discussion

We developed a validated psychometric scale consisting of 17 items and two domain factors, including perception (RDU and AMS) and attitude (PRO and IPP) toward AMR and AMS. Psychometric instruments have been widely used to assess healthcare professionals’ competencies in areas such as radiation safety and interprofessional communication [36, 37]. This supports the methodological approach used in developing the PATARAS scale, which similarly measures attitudes and perceptions within a healthcare education context. The PATARAS instrument features a multi-domain structure with a hierarchical model comprising two domains and four subdomains. Confirmatory factor analysis (CFA) confirmed the scale's acceptable validity and reliability (Tables 2 and 3).

Pharmacists must have excellent competence related to antibiotics use. Data on pharmacy students’ perceptions and attitudes toward AMR and AMS can be used to strengthen pharmacy education. Specifically, the PATARAS findings highlight key areas, such as professional responsibility (PRO) and inappropriate practices (IPP), that require greater emphasis in teaching and assessment. Educators can use this information to design targeted interventions that reinforce responsible antibiotic use and enhance students’ understanding of stewardship principles. The pandemic exacerbated the AMR problem, mainly by the irrational use of antiviral, antihelmintic, antimalarial, and especially macrolide antibiotics [38]. Our results demonstrate significant positive relationships between RDU and PRO, RDU and IPP, and AMS and PRO, and a negative correlation between AMS and IPP. These findings indicate that a higher perception of AMS lowers IPP. This suggests that a higher perception of AMS is associated with lower levels of inappropriate practices. These findings are further supported by the structural model presented in Table 4. A strong association between AMS and professional role suggests that greater awareness of AMS enhances students’ commitment to their professional responsibilities [39]. While RDU also supports professional attitudes, it may not always translate into appropriate practice. Some aspects of RDU education have been linked to inappropriate prescribing behaviors, highlighting a gap between knowledge and real-world application [40]. This finding suggests that a lower perception of rational drug use is associated with higher levels of inappropriate practices, such as dispensing antibiotics over the counter [40]. In contrast, AMS is associated with a reduction in such inappropriate practices, emphasizing its importance in promoting responsible antibiotic use [41]. These findings support the structural validity of the PATARAS scale and reinforce that perception-based education plays a critical role in shaping professional behavior in pharmacy students [42]. A meta-analysis of the effects of pharmacist-led AMS programs on inappropriate antibiotic prescribing and management is in agreement with our findings [43]. In the pharmacy education field, perceptions, and attitudes toward antibiotics, AMR, and AMS can be predicted with many questionnaire items. Although in this study only two perception subdomains (RDU and AMS) were used to predict attitudes, the findings indicate that these subdomains effectively captured student pharmacists’ attitudes toward AMR and AMS.

Previous studies evaluated pharmacists’ knowledge, perception, and attitudes toward antibiotic use; however, these studies did not include a theoretical framework, specifically for AMR and AMS, in their assessments [14, 44, 45]. We argue that any new instrument must cover all theoretical concepts related to AMR and AMS in the pharmacists’ context. Support for the content validity of the psychometric scale used in our study was based on a theoretical framework. During the psychometric scale construction, lecturers, and pharmacy professionals were invited to modify, eliminate, and revise instrument items. The choice of which items to include in the final instrument is critical, as poorly developed instruments might lead researchers to draw incorrect conclusions about the examined issue [46, 47]. The main reason for drawing incorrect conclusions is that some previous studies did not use proper psychometric measurements such as CFA in this study. Moreover, studies linking perceptions and attitudes related to antibiotic use, AMS, and AMR among pharmacy students are scarce [5–8]. Only three Indonesian studies [48–50] evaluated the perception and attitude toward antibiotic use, AMS, and AMR. Those studies did not investigate IPP and focused on healthcare practitioners, not pharmacy students. A previous study in China reported that medical students had good perceptions and attitudes, but did not exhibit good practice toward prescribing antibiotics [51]. The same trend was found in a study involving B.Sc pharmacy students in Trinidad and Tobago. The authors reported that the students had good perceived knowledge on antibiotic use and resistance but their knowledge was not translated into practice [45, 52]. A study of pharmacy students from Malaysia also found that most students had a high level of understanding of AMR, but their attitudes did not correlate well [53]. In our questionnaire, inappropriate practice items (IPP 2 and IPP 3) were related to self-medication and drug stock, where many students believed that antibiotics could be given to meet patient needs, despite the fact that knowledge and perceptions about rational drug use were expected to reduce this problem. A significant contributor to the higher incidence of antibiotic self-medication, even among students, was having antibiotic stocks at home [54, 55].

This research has some limitations. Further development testing of the instrument is recommended for various healthcare students, i.e. medical, nurse, and public health because our study was limited to pharmacy students.

## Conclusion

This study indicates that the PATARAS questionnaire is a valid and reliable instrument to measure perceptions and attitudes toward AMR and AMS in Indonesia. The PATARAS questionnaire exhibited acceptable-to-good psychometric properties, based on construct validity, reliability, and an acceptable threshold, based on CFA. The findings highlight the importance of strengthening antimicrobial-related education at the undergraduate level and offer practical insights for pharmacy educators and universities seeking to assess and enhance students’ competencies in AMR and AMS. Moreover, the PATARAS instrument may serve as a useful tool for evaluating the effectiveness of educational interventions designed to improve students’ attitudes and understanding of AMS principles. Overall, the study reinforces the critical role of perception-based education in shaping future pharmacists’ professional attitudes and behaviors toward responsible antibiotic use.

## Supplementary Information

Supplementary Material 1.

## Data Availability

Data is provided within the manuscript or supplementary information files.
