# Supplementary material for: Development and validation of a psychometric scale for assessing pharmacy students’ perceptions and attitudes toward antimicrobial resistance and antimicrobial stewardship in Indonesia: the PATARAS study
Source: BMC Med Educ. 2025 May 30;25:805. doi: 10.1186/s12909-025-07375-5 (PMC12125818; doi:10.1186/s12909-025-07375-5)
Supplement: Supplementary file 1 — Supplementary Material 1. [file 12909_2025_7375_MOESM1_ESM.docx]

**Appendix 1** Content Validity Index (CVI) of the 20-items draft PATARAS from 5 experts

| **Variable Construct** | **Panel 1** | | **Panel 2** | | **Panel 3** | | **Panel 4** | | **Panel 5** | | **Expert in Agreements** | **I-CVI Score** | **S-CVI Score** |
| --- | --- | --- | --- | --- | --- | --- | --- | --- | --- | --- | --- | --- | --- |
|  | **n** | **Code** | **n** | **Code** | **n** | **Code** | **n** | **Code** | **n** | **Code** |  |  |  |
| RDU1 | 4 | 1 | 4 | 1 | 4 | 1 | 4 | 1 | 4 | 1 | 5 | 1.00 | 1 |
| RDU2 | 4 | 1 | 4 | 1 | 4 | 1 | 3 | 1 | 3 | 1 | 5 | 1.00 | 1 |
| RDU3 | 4 | 1 | 4 | 1 | 4 | 1 | 3 | 1 | 3 | 1 | 5 | 1.00 | 1 |
| RDU4 | 4 | 1 | 4 | 1 | 4 | 1 | 4 | 1 | 4 | 1 | 5 | 1.00 | 1 |
| RDU5 | 4 | 1 | 3 | 1 | 4 | 1 | 4 | 1 | 4 | 1 | 5 | 1.00 | 1 |
| RDU6 | 4 | 1 | 4 | 1 | 4 | 1 | 4 | 1 | 4 | 1 | 5 | 1.00 | 1 |
| RDU7 | 4 | 1 | 4 | 1 | 4 | 1 | 4 | 1 | 4 | 1 | 5 | 1.00 | 1 |
| AMS1 | 4 | 1 | 4 | 1 | 4 | 1 | 4 | 1 | 4 | 1 | 5 | 1.00 | 1 |
| AMS2 | 3 | 1 | 4 | 1 | 4 | 1 | 3 | 1 | 3 | 1 | 5 | 1.00 | 1 |
| AMS3 | 4 | 1 | 4 | 1 | 4 | 1 | 3 | 1 | 3 | 1 | 5 | 1.00 | 1 |
| PRO1 | 4 | 1 | 4 | 1 | 4 | 1 | 4 | 1 | 4 | 1 | 5 | 1.00 | 1 |
| PRO2 | 3 | 1 | 3 | 1 | 4 | 1 | 4 | 1 | 4 | 1 | 5 | 1.00 | 1 |
| PRO3 | 4 | 1 | 4 | 1 | 4 | 1 | 3 | 1 | 3 | 1 | 5 | 1.00 | 1 |
| PRO4 | 4 | 1 | 4 | 1 | 4 | 1 | 3 | 1 | 3 | 1 | 5 | 1.00 | 1 |
| IPP1 | 4 | 1 | 4 | 1 | 3 | 1 | 4 | 1 | 4 | 1 | 5 | 1.00 | 1 |
| IPP2 | 3 | 1 | 4 | 1 | 4 | 1 | 4 | 1 | 4 | 1 | 5 | 1.00 | 1 |
| IPP3 | 4 | 1 | 3 | 1 | 4 | 1 | 3 | 1 | 3 | 1 | 5 | 1.00 | 1 |
| IPP4* | 2 | 0 | 3 | 1 | 2 | 0 | 3 | 1 | 2 | 0 | 2 | 0.40 | 0 |
| IPP5* | 2 | 0 | 3 | 1 | 4 | 1 | 2 | 0 | 2 | 0 | 2 | 0.40 | 0 |
| IPP6* | 2 | 0 | 2 | 0 | 2 | 0 | 3 | 1 | 3 | 1 | 2 | 0.40 | 0 |
| Mean |  | 0.850 |  | 0.950 |  | 0.900 |  | 0.950 |  | 0.900 | Summary | 18.20 | 17.00 |
| Average proportion of items judged as relevance across the 5 experts | | | | | | | | | | | Average | 0.91 | 0.85 |

I-CVI, item level content validity index; S-CVI, scale level content validity index

*removed item
